# βC1, pathogenicity determinant encoded by Cotton leaf curl Multan betasatellite, interacts with calmodulin-like protein 11 (Gh-CML11) in *Gossypium hirsutum*

**DOI:** 10.1371/journal.pone.0225876

**Published:** 2019-12-03

**Authors:** Hira Kamal, Fayyaz-ul-Amir Afsar Minhas, Diwaker Tripathi, Wajid Arshad Abbasi, Muhammad Hamza, Roma Mustafa, Muhammad Zuhaib Khan, Shahid Mansoor, Hanu R. Pappu, Imran Amin

**Affiliations:** 1 National Institute for Biotechnology and Genetic Engineering, Faisalabad, Pakistan; 2 Pakistan Institute of Engineering and Applied Sciences (PIEAS), Nilore, Islamabad, Pakistan; 3 Department of Plant Pathology, Washington State University, Pullman, WA, United States of America; 4 Department of Biology, University of Washington, Seattle, WA, United States of America; Institute for Sustainable Plant Protection, C.N.R., ITALY

## Abstract

Begomoviruses interfere with host plant machinery to evade host defense mechanism by interacting with plant proteins. In the old world, this group of viruses are usually associated with betasatellite that induces severe disease symptoms by encoding a protein, βC1, which is a pathogenicity determinant. Here, we show that βC1 encoded by Cotton leaf curl Multan betasatellite (CLCuMB) requires *Gossypium hirsutum* calmodulin-like protein 11 (Gh-CML11) to infect cotton. First, we used the *in silico* approach to predict the interaction of CLCuMB-βC1 with Gh-CML11. A number of sequence- and structure-based *in-silico* interaction prediction techniques suggested a strong putative binding of CLCuMB-βC1 with Gh-CML11 in a Ca^+2^-dependent manner. *In-silico* interaction prediction was then confirmed by three different experimental approaches: The Gh-CML11 interaction was confirmed using CLCuMB-βC1 in a yeast two hybrid system and pull down assay. These results were further validated using bimolecular fluorescence complementation system showing the interaction in cytoplasmic veins of *Nicotiana benthamiana*. Bioinformatics and molecular studies suggested that CLCuMB-βC1 induces the overexpression of Gh-CML11 protein and ultimately provides calcium as a nutrient source for virus movement and transmission. This is the first comprehensive study on the interaction between CLCuMB-βC1 and Gh-CML11 proteins which provided insights into our understating of the role of βC1 in cotton leaf curl disease.

## Introduction

Family *Geminiviridae* is one of the largest groups of plant viruses. Members in this family possess circular, single-stranded (ss) DNA as their genome, and infect economically important crops such as cotton, cassava, grains, maize, and vegetables [1]. On the basis of genome organization, vector transmission, genome wide pairwise sequence identity and host range, this family is classified into nine genera that include *Becurto-*, *Begomo-*, *Capula-*, *Curto-*, *Eragro-*, *Grablo-*, *Mastre-*, *Topocu- and Turncurto-virus*. *Begomovirus* is the largest and economically important genus of this family [2]. Begomoviruses infect dicotyledonous plant species in tropical and subtropical regions and are found in both New World (NW) and Old World (OW). The genus represents the largest number of viruses possessing more than 320 species under the family Geminiviridae [2]. It possesses either monopartite (DNA-A) or bipartite genome (DNA-A and DNA-B) of approximately 2.6–2.8 kb size that are different from each other except the region of almost 200 nucleotides (common region; CR) present within the intergenic region (IR) [3]. However, genome organization in monopartite viruses possess all proteins that are sufficient for viral replication, encapsidation and transmission across the different hosts [4]. Most of the OW monopartite begomoviruses are associated with satellite DNAs referred to as betasatellite (genus Betasatellite, family *Tolecusatellitidae*) and alphasatellite (family *Alphasatellitidae*) [5–9]. Satellites are the small molecules with the size of ~1.4 kb, and are dependent on helper virus for their replication, virus packaging (encapsidation), intracellular movement and transmission. These satellite molecules associated with begomovirus were discovered with the identification of *Tomato leaf curl virus* (ToLCV) [3]. Betasatellites encode βC1 protein which is a pathogenicity determinant while the role of alphasatellite is not fully known [10].

Cotton (*Gossypium hirsutum*) is a major cash crop of Pakistan and contributes up to 60% of foreign exchange earnings [11]. In Punjab province, cotton is grown on approximately 2.5 million hectares [12]. Cotton leaf curl disease (CLCuD) is one of the major constraints to the production of cotton in the Indian sub-continent which is caused by several begomoviruses in association with a specific betasatellite known as Cotton leaf curl Multan betasatellite (CLCuMuB). A number of distinct begomovirus species were shown to be associated with the disease, the most important of which are *Cotton leaf curl Multan virus* (CLCuMuV) and *Cotton leaf curl Kokhran virus*-Burewala strain (CLCuKoV-Bur) [13]. These viruses are poorly infectious to cotton and require CLCuMuB to cause CLCuD [11]. βC1 is the only protein encoded by Betasatellite and was shown to be involved in the induction of disease symptoms [14, 15], viral DNA movement [16], and also acts as a RNA silencing suppressor (RSS; a plant defense mechanism which target transcripts of invading viruses and generated through dsRNA) [17, 18], forms homo multimeric complexes in plants [19], interferes with host plant gene expression and was also identified to interact with a number of host factors [20]. Among various host proteins, βC1 was found to interact with the calmodulin protein family which acts as a modulator of calcium signaling in plants. βC1 encoded by begomovirus Tomato yellow leaf curl China β-satellite (TYLCCNB) upregulates calmodulin-like (CML) protein in *Nicotiana benthamiana* (NbrsgCaM) resulting in repression of RNA-dependent RNA polymerase 6 (RDR6), that contributes to the antiviral response in plants [21]. Transcription activator protein (TrAP; C2) encoded by begomovirus *Tomato golden mosaic virus* (TGMV) also binds to and upregulates CML protein rgsCaM in *Arabidopsis thaliana* and *N*. *benthamiana*. This C2 interaction over expresses rgsCaM in the nucleus that results into higher susceptibility to the TGMV infection [22].

Calmodulin (CaM) and CML protein belongs to the calcium binding protein family in plants that possess EF-hand (helix-loop-helix) like structure. EF-hand is more likely spread thumb and forefinger in which two alpha helices are linked with a loop region that may attain bound and unbound state of calcium ion to modulate the intracellular Ca^+2^ concentration. This apo (bound) and holo (free) state of Calmodulin protein is required for energy metabolism, and plays a role in host defense system [23]. The sequence and structure of CaM and CML proteins vary in *A*. *thaliana*, rice, and tobacco especially from linker (middle part connecting N- and C- lobe) region in their structures. This connecting region produces more flexibility in CML as compare to CaM using different conformations in plants, which extends strong interaction with neighboring proteins [24]. Moreover, it has been observed that CML proteins have evolved earlier than CaM [25]. In previous reports, several pathogens such as bacteria, fungi and viruses interact with different members of CML protein in different cell compartments, indicating a strong relation between Ca^+2^ ion channels and pathogens in plasma membrane and the nucleus [26]. For example, CML43 and its orthologs in tomato enhances the production of avirulent strain of *Pseudomonas syringae* to cause bacterial infection in tomato, and CML9 functions mainly in response to avirulent pathogens [27, 28]. During fungal infection by *Verticillium dahliae*, calcium influx is also upregulated which disturbs calcium-based responsive genes in cotton. To counteract, the Gh-CML11 in *G*. *hirsutum* can also bind with transcription factor MYB108 which forms a positive loop and enhances tolerance against this fungal attack [29]. All these studies suggest that the CML protein has different mechanisms of action with different types of pathogens and many CML proteins are still in need to be characterized with respect to their structure and function [30, 31].

Limited reports are available on the role of CaM and CML proteins in plant virus infection. CML38 in *A*. *thaliana* binds to HC-Pro to enhance *Potyvirus*, *Turnip mosaic virus* (TuMV) replication. The same viral protein (HC-Pro) that acts as RSS from another *Potyvirus Tobacco etch virus* (TEV) associates directly with rgsCaM from tobacco, acting as an endogenous suppressor for HC-Pro [32]. Binding of rgsCaM with HC-Pro reduces its abundance in plant cells which can facilitate viral infection to spread [32]. Therefore, plant viruses deregulates Ca^+2^ channels using Ca^+2^ dependent protein such as CaM to aid virus replication and its transcription in a host [33]. All these studies showed diverse roles of CML proteins during pathogen attack.

Bioinformatic approaches provide useful tools to investigate the role of CaM and CML members in plant-pathogen interactions. These *in-silico* approaches rely on several sequential steps to investigate protein-protein interaction (PPI) such as sequence-based method that extracts information from unique sequence motifs to build protein secondary structure [34], binding sites detection methods associated with the sequence, and 3D structures that predict interfacial residues in close proximity [35]. Aa a final step, the interface prediction method retrieves the information from experimentally determined interacting residues using machine learning methods and utilizes trained models to identify interacting residues of a query protein [36]. This multi-pronged approach based on sequence conservation analysis, energetics and binding site can be used to predict the interaction between virus and host proteins such as geminivirus proteins and their interacting partners in cotton and furthermore, to localize the residues in the interacting domain that are responsible for their binding affinity.

Here, we extend our analyses on CML protein in cotton that binds with one of the known begomovirus-associated satellites, CLCuMB. We report that CLCuMB-encoded βC1 protein binds to Gh-CML11 in *G*. *hirsutum* for virus pathogenicity and symptom enhancement. We first used a suite of *in-silico* methods to predict the putative interaction and its strength between these two proteins. Moreover, CaM binding (CaMB) motifs in CLCuMB-βC1 suggested calcium dependent interaction with Gh-CML11. This interaction prediction was further validated by three independent experimental approaches: yeast two-hybrid (Y2H), pull-down assay and bimolecular fluorescence complementation (BiFC). Taken together, our findings provide a better insight into the cotton-begomovirus interactions underlying the CLCuD.

## Materials and methods

### Bioinformatics analysis of Gh-CML11 and CLCuMB-βC1

Various bioinformatics tools were used for predicting the interaction between Gh-CML11 and CLCuMB-βC1 proteins, their binding affinity and binding sites. We first predicted the structures of Gh-CML11 and CLCuMB-βC1 using I-TASSER [37] and subsequently used as input to structure-based bioinformatics methods. CaMELS [38], PPA-Pred [39], PRISM [40] and PRODIGY [41] were used for interaction and binding affinity prediction. Meanwhile, CaM/CML binding motifs were identified in CLCuMB-βC1 sequence using reported data [42], CaM binding (CaMB) database including Calmodulin Target Database [43], Calmodulation database and meta-analysis predictor [44]. High binding affinity and CaMB motifs proposed a strong interaction between these two proteins. Further bioinformatics analysis to identify the binding site(s) at residue level was carried out using BSpred [45], NSPHOMPPI [46], PSIVER [47], PredictProtein [48], PPiPP [49], CPORT [50], ZDOCK [51], Docking2 at ROSETTA [52] and PAIRpred [53]. All these methods predicted different binding regions and we combined the results using an ensemble heuristic based on majority voting. Myristylation signal was identified in Gh-CML11 using Myristylator [54] and NMT server [55].

### Plant lines and sample processing

For *in-planta* interaction study, wild type and transgenic lines of *N*. *benthamiana* seeds with the CFP-H2B marker were grown in pots containing Sunshine Mix LC1 in a greenhouse with 16 h light/8 h dark cycle. A confirmed clone for CLCuMB (AM774307), was used for amplification of βC1. CML protein was isolated from a CLCuD resistant cotton variety, UA222. RNA was obtained from cotton leaves using RNeasy Plant Mini Kit (Qiagen, Netherlands) following the manufacturer’s instructions. Purified RNA was then reverse transcribed to generate cDNA using a RevertAid first strand cDNA synthesis kit (Thermo Scientific, USA) with an oligo (dT) primer. RT-PCR was performed to amplify CLCuMB-βC1 and Gh-CML11 genes using Phusion high-fidelity DNA polymerase (Thermo Scientific, USA) and attB sequence-flanked specific primers, listed in S1 Table. For PCR amplification only 1–2 μl cDNA (50–150 ng/ μl) was used to a master-mix of total 50 μl reaction. It contained 1M MgSO_4_ (2 mM final conc.), F+R primers (0.5 μM each), dNTPs (0.15 Mm final conc.), 10X PCR buffer, *pfx* polymerase (1 U) and water to make up the volume upto 50 μl. The PCR profile consist of 1 initial denaturation cycle was set at 95 ^o^C for 1 minute, followed by 35 cycles of denaturation step at 95 ^o^C for 30 sec, annealing step at 58–60 ^o^C for 30 sec and extension step at 72 ^o^C for 45 sec (according to template size and polymerase efficiency). Then final extension was set at 72 ^o^C for 5 minutes. All amplified products were separately cloned into pENTR-D-TOPO vector (Invitrogen, USA) and positive clones were confirmed by sequencing using M13 forward and reverse primers, and gene-specific primers.

### Yeast two hybrid (Y2H) and Pull down assays

For Y2H, entry clones from Gateway^®^ cloning system were recombined into the binary vector pEZY202 (Addgene) and pEZY45 (Addgene) [56]. Lithium acetate yeast transformation procedure as described [57, 58] was followed using yeast (*Saccharomyces cerevisiae*) strain EGY48 possessing pSH18-34 marker. Screening for positive interaction was performed on minimal SD Base/Gal/Raf with triple dropout medium–His/-Trp/-Ura (TDO) and Quadruple dropout medium -His/-Leu/-Trp/-Ura (QDO). Background was eliminated using 3-Amino-1,2,4-triazole (3-AT) ranging in 10-40mM serial dilution for positive interaction only between bait and pair.

The maltose binding protein (MBP) pull down assay was performed following the protocol as described previously [59]. Gateway entry clones were recombined into pull down destination vectors pMAL-c2X and pDEST15 (Invitrogen, USA). Protein samples expressed in *E*.*coli* BL21 (DE3) strain were purified by sonication and purified products were eluted after two to three washes. For Western blotting, samples were separated on sodium dodecyl sulfate-polyacrylamide gel electrophoresis (SDS-PAGE) and incubated with monoclonal anti-GST antibody (primary) which was subsequently probed with secondary antibody goat HRP-conjugated anti-rabbit IgG (Bio-Rad, USA). Positive signals were developed on x-ray film using ECL method based on Versa Doc Imaging System (Bio-Rad, USA) following the manufacturer’s instructions.

### Bimolecular fluorescence complementation (BiFC) study

Genes were cloned from entry vector into BiFC binary vectors pSITE-cEYFP-C1+pSITE-nEYFP-C1 (ABRC; Ohio) using LR Clonase^TM^ enzyme mix. *Agrobacterium* transformation, infiltration and confocal microscopy were done by following the protocol as described [60]. Agroinfiltrated leaves containing host and viral proteins were studied after 24-48hrs incubation under a confocal microscope. Transgenic lines of *N*. *benthamiana* possessing CFP-H2B marker [61], was used to inspect under YFP and CFP fluorescent markers. A minimum of three leaves were evaluated, and images were acquired using TCS SP8 X microscope (Leica Germany) at 20X dry, 40X dry and 63X oil for fine detail images and LAS X software was used to analyze the data.

## Results

### *In silico* Gh-CML11 and CLCuMB-βC1 interaction prediction

**Fig 1:**
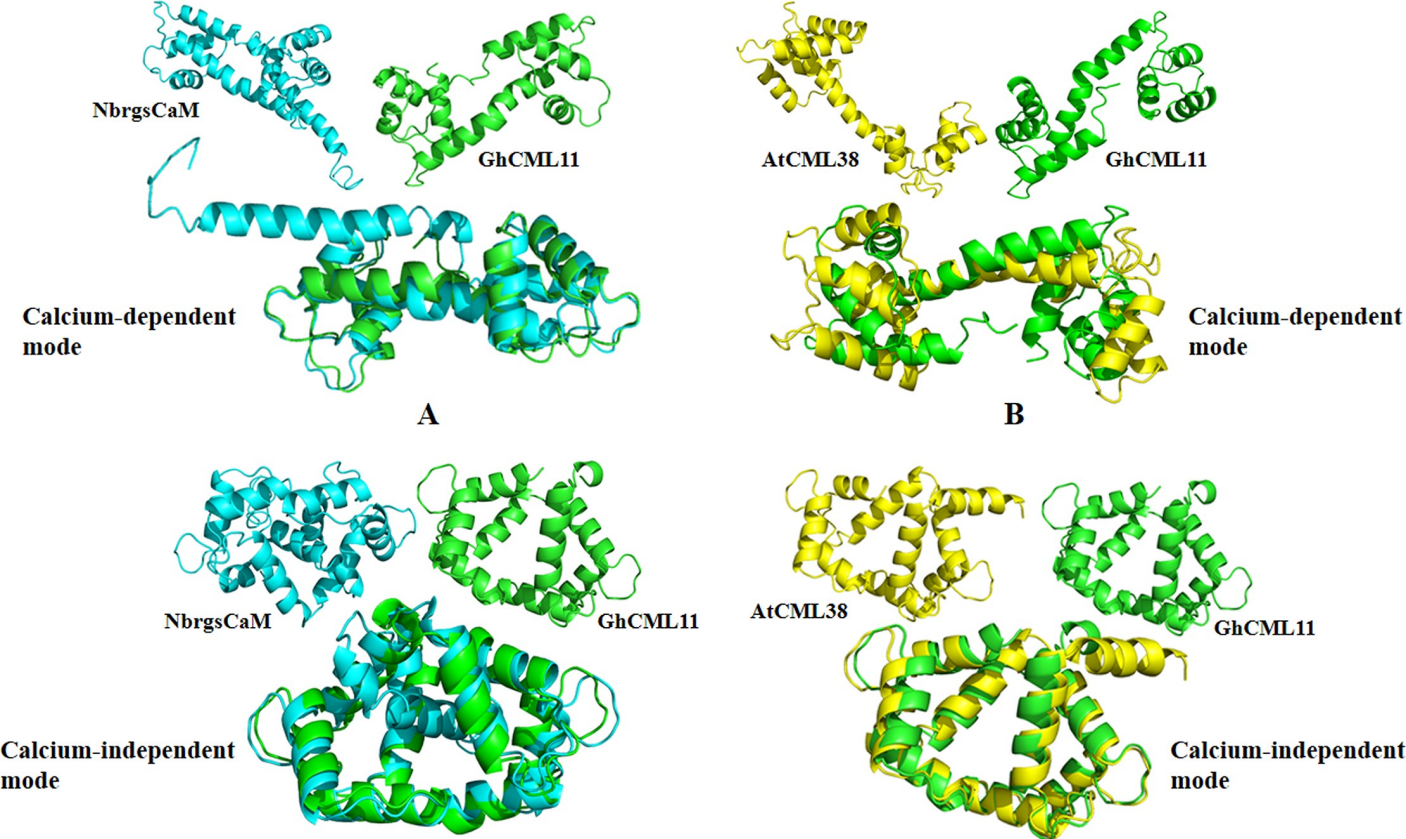
Structural comparison of Gh-CML11 with other CML members in *Nicotiana benthamiana* and *Arabidopsis thaliana*. Calcium dependent and calcium independent structural variations for Gh-CML11 protein with **A.** NbrsgCaM and **B.** AtCML38.

Sequence and structure analysis were performed before identifying CLCuMB-βC1 interaction with CML11 based on molecular techniques. Previous studies have shown and rgsCaM from *N*. *benthamiana* and CML38 from *A*. *thaliana*, respectively interacts with geminiviruses (TGMV, TYLCCNV) and potyvirus (TuMV) as described [21, 22, 62]. In the current study, sequence analyses showed that Gh-CML11 protein shares only 24% identity with CaM protein in NbrsgCaM and 26% identity with AtCML38 S2 Table. However, structure alignment shows rgsCaM from *N*. *benthamiana* forms a long linker region in calcium-dependent manner and shares structure identity with Gh-CML11 under RMSD of 1.68 Å, while in calcium-independent manner shares 5.32 Å RMSD (Fig 1A). Low RMSD indicates high structure identity and vice versa. In case of AtCML38 and GhCML11, both protein complexes possess low structural identity during calcium-dependent manner showing RMSD of 6.78 Å and 1.24 Å RMSD during calcium-independent manner (Fig 1B). AtCML38 consist of 4 α-helices at N-lobe, forming 3 EF-hands connected with C-lobe through a long α-helix linker part. Gh-CML11 consists of 2 EF-hands present on each domain connected with a middle linker region (Fig 1B). This data represents CML protein in *G*. *hirsutum* possess variations with other CaM/CML protein members in *N*. *benthamiana* and *A*. *thaliana* at sequence and structure level. In geminiviruses, TYLCCNB was shown to interact with CML protein in *N*. *benthamiana*, but TYLCCNB-βC1 shares only 30% identity with CLCuMB-βC1 S2 Table. This sequence to structure analysis data suggested that CLCuMB-βC1 may or may not have the binding ability with Gh-CML11 protein which is in need to be investigated independently for CLCuMV role in the presence of calmodulin like protein.

As Protein Data Bank (PDB) structures for both virus and host proteins were not available online, 3D structures for both of them were predicted using I-TASSER [37]. I-TASSER predicted five models for both proteins and the best model was chosen based on a higher value of C-score (confidence score). C-score for Gh-CML11 ranged from -0.27 to -2.58 and CLCuMB-βC1 had a -1.68 to 3.89 score. The model with the lowest negative value shows more stable structure and was selected for further study. Moreover, to determine the potential interaction, a number of methods were used with respect to binding affinity where ΔG (Gibbs free energy) represents binding energy or equilibrium constant. For example, when protein A binds with protein B in its neighbor forms a complex AB due to strong relationship. It will possess higher negative value (Gibbs free energy, ΔG<0) due to less conformational changes. Similarly, due to strong binding, this AB complex possess high equilibrium constant in terms of change in Gibbs free energy (ΔΔG) [ΔΔG_AB_ = ΔG_AB_-(ΔG_A_+ΔG_B_)]. In simple notion, ΔΔG determines either interaction is more stable (ΔG<0) or less stable (ΔG>0). Initially interaction was determined with CaMELS [38], a sequence-based method that can be used to predict the propensity of interaction of a protein with CaM and CML proteins as well as identification of the CaM or CML binding sites on these proteins. It produces a score of 5.04 for interaction of CLCuMB-βC1 S3 Table. This high positive value corresponds to a higher binding affinity and a possible interaction between these proteins. This score was further cross checked with another sequence-based computational method PPA-pred [39] which directly predicts binding affinity between two proteins in terms of change in Gibbs free energy (ΔΔG). Based on functional relevance of a protein complex, the PPA-Pred predicted value of ΔΔG for the possible interaction between CLCuMB-βC1 and Gh-CML11 was -11.65 kcal/mol that also indicated a high probability of interaction. Though, sequence-based methods possess some limitations such as these methods are not able to assess energy for a complex during different modes of three dimensional structures of both interacting proteins. For this reason, structure-based approach PRISM [40] and PRODIGY [41] were used to predict the binding affinity of these proteins. The PRISM determined ΔΔG value was -18.32 kcal/mol and PRODIGY detected -41.2 kcal/mol between CLCuMB-βC1 and Gh-CML11 S3 Table, showing a strong binding energy between them. Using sequence and structure-based methods, a high ΔΔG value indicated a higher possibility of interaction between CLCuMB-βC1 and Gh-CML11.

**Fig 2:**
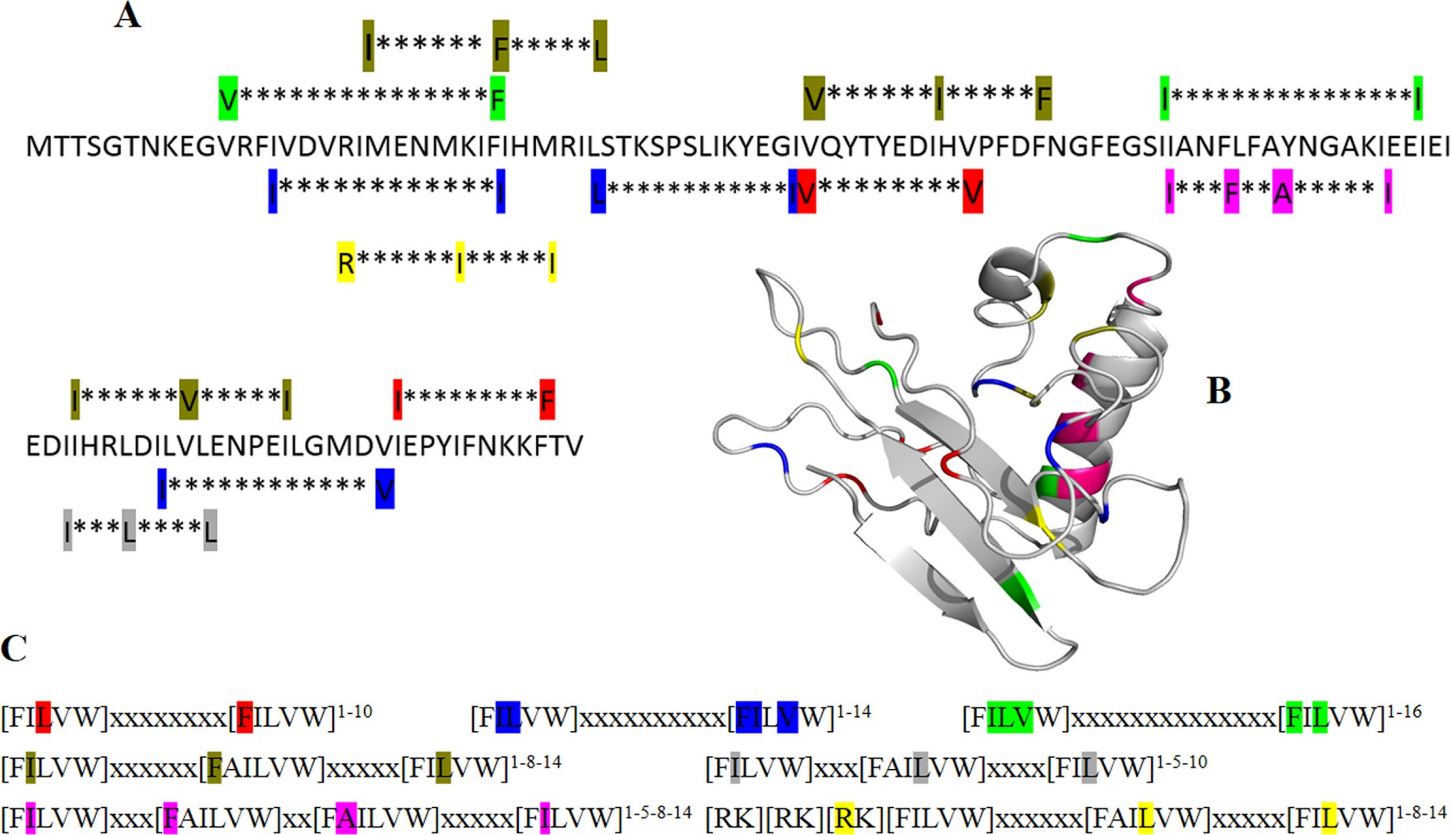
Identification of CaMB motifs in CLCuMB-βC1 sequence and structure. **A.** Selected CaM binding motifs are highlighted in CLCuMB-βC1 protein showing potential binding capacity with Gh-CML11. **B.** Deletion of CaMB motif 1-5-8-14 reduces ΔΔG value suggesting its role in binding at positions 66–79 in CLCuMB-βC1 α-helix (highlighted with purple color). **C.** Most known canonical CaMB motifs listed here are present in CLCuMB-βC1, indicating its potential interaction with calmodulin like protein in Ca^+2^-dependent manner.

Short linear amino acid motifs termed as CaM binding motifs (CaMBM) are useful in identifying all possible CaM-binding proteins in plants during biotic and abiotic stresses. The CaMB motifs in CLCuMB-βC1 were also another source of interaction prediction between these two proteins. Using databases such as Calmodulin Target database, Calmodulation database and meta-analysis predictor [42–44], almost 66 CaMB motifs either in canonical or noncanonical form were identified in CLCuMB-βC1 protein (Table 1). It has been observed that these motifs are present in overlapping form throughout the whole sequence and structure (Fig 2A). Motif 1-5-8-14 at position 66–79 in CLCuMB-βC1 is involved in α-helix formation shown in purple Fig 2B, and deletion of this motif reduces the predicted ΔΔG value indicating its role with Gh-CML11 among all reported hydrophobic CaMB motifs (Fig 2C). Due to these extensive motifs in CLCuMB-βC1 sequence, it is possible that it can bind with CML protein and a majority of these canonical CaMB forms indicated that CLCuMB-βC1 potentially modulates binding with Gh-CML11 protein in a Ca^+2^-dependent manner.

**Table 1:** Possible canonical CaM binding motifs in CLCuMB-βC1.

Motif	Sequence	Residue	Motif	Sequence	Residue	Motif	Sequence	Residue
1–10	VRIMENMKIF	17–26	1–12	VLENPEILGMDV	95–106	1–16	IKYEGIVQYTYEDIHV	40–55
1–10	ILSTKSPSLI	31–40	1–12	ILGMDVNEPYVF	101–112	1–16	VPFDFNGFEGSIIANF	55–70
1–10	VQYTYEDIHV	46–55	1–14	FIVDVRIMENMKIF	13–26	1–16	FDFNGFEGSIIANFLF	57–72
1–10	IHVPFDFNGF	53–62	1–14	IVDVRIMENMKIFI	14–27	1–16	IANFLFAYNGAKIEEI	67–82
1–10	FDFNGFEGSI	57–66	1–14	IMENMKIFIHMRIL	19–32	1–16	FAYNGAKIEEIEIEDI	72–87
1–10	FEGSIIANFL	62–71	1–14	FIHMRILSTKSPSL	26–39	1–16	IEEIEIEDIVHRLDIL	79–94
1–10	FLFAYNGAKI	70–79	1–14	IHMRILSTKSPSLI	27–40	1–16	IVHRLDILVLENPEIL	87–102
1–10	IEEIEIEDIV	79–88	1–14	LSTKSPSLIKYEGI	32–45	1–16	LDILVLENPEILGMDV	91–106
1–10	IEIEDIVHRL	82–91	1–14	IKYEGIVQYTYEDI	40–53	1–16	LENPEILGMDVNEPYV	96–111
1–10	IEDIVHRLDI	84–93	1–14	VQYTYEDIHVPFDF	46–59	1–16	ILGMDVNEPYVFNKKF	101–116
1–10	IVHRLDILVL	87–96	1–14	IHVPFDFNGFEGSI	53–66	1-5-10	IHVPFDFNGF	53–62
1–10	ILVLENPEIL	93–102	1–14	FDFNGFEGSIIANF	57–70	1-5-10	FEGSIIANFL	62–71
1–10	LGMDVNEPYV	102–111	1–14	FNGFEGSIIANFLF	59–72	1-5-10	IEDIVHRLDI	84–93
1–12	IVDVRIMENMKI	14–25	1–14	IIANFLFAYNGAKI	66–79	1-5-10	IVHRLDILVL	87–96
1–12	VDVRIMENMKIF	15–26	1–14	LFAYNGAKIEEIEI	71–84	1-5-10	LGMDVNEPYV	102–111
1–12	VQYTYEDIHVPF	46–57	1–14	IEIEDIVHRLDILV	82–95	1-5-8-14	IIANFLFAYNGAKI	66–79
1–12	VPFDFNGFEGSI	55–66	1–14	VHRLDILVLENPEI	88–101	1-8-14	IMENMKIFIHMRIL	19–32
1–12	FNGFEGSIIANF	59–70	1–14	ILVLENPEILGMDV	93–106	1-8-14	LSTKSPSLIKYEGI	32–45
1–12	LFAYNGAKIEEI	71–82	1–16	VRFIVDVRIMENMKIF	11–26	1-8-14	VQYTYEDIHVPFDF	46–59
1–12	IEIEDIVHRLDI	82–93	1–16	VRIMENMKIFIHMRIL	17–32	1-8-14	FNGFEGSIIANFLF	59–72
1–12	IEDIVHRLDILV	84–95	1–16	IFIHMRILSTKSPSLI	25–40	1-8-14	IIANFLFAYNGAKI	66–79
1–12	LDILVLENPEIL	91–102	1–16	ILSTKSPSLIKYEGIV	31–46	1-8-14	VHRLDILVLENPEI	88–101

### Bioinformatics study for interface prediction

**Fig 3:**
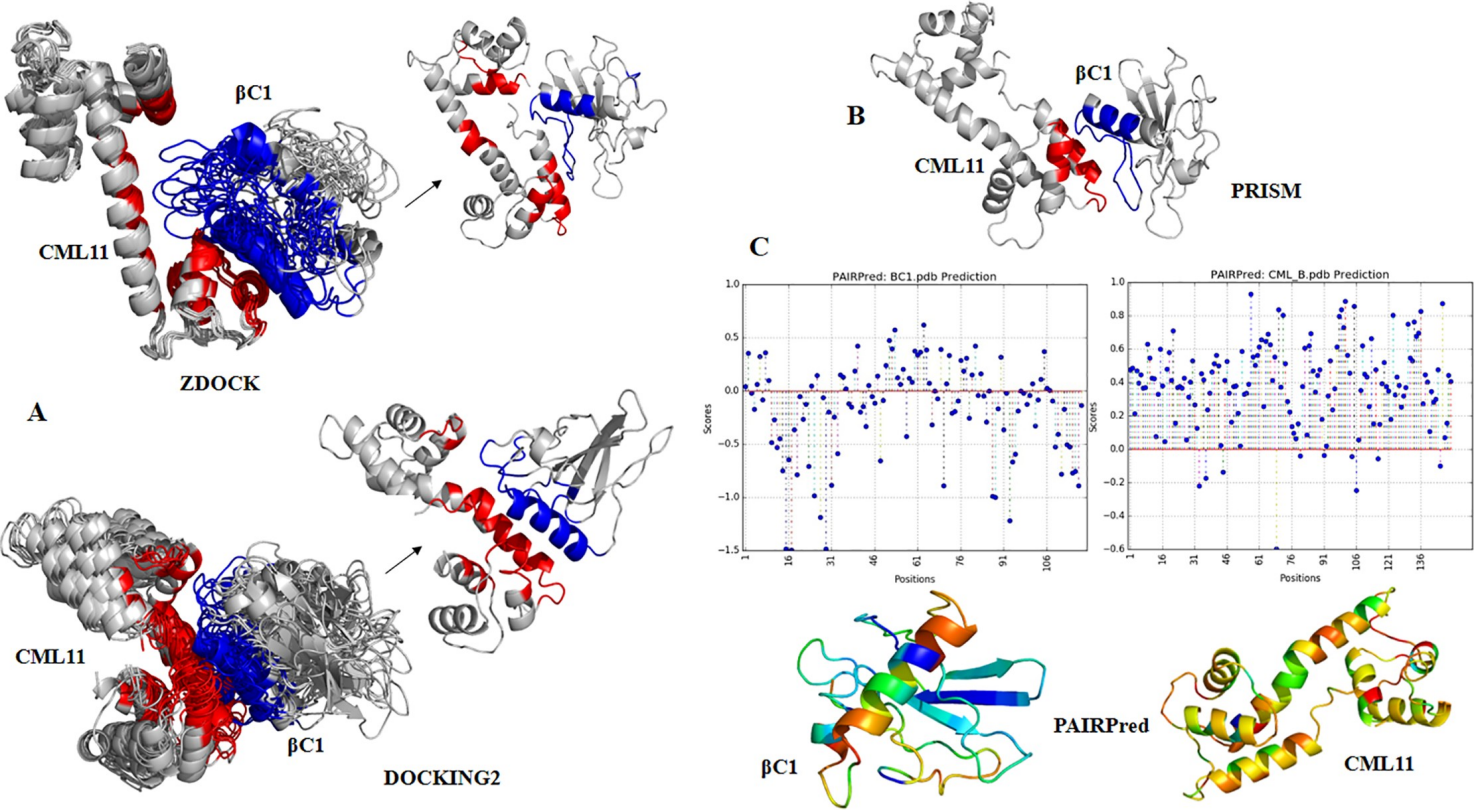
Interaction prediction using protein docking methods and machine learning method using 3D structures of CLCuMB-βC1 and Gh-CML11 protein. **A.** Top ten models predicted by docking methods ZDOCK and DOCKING2 were aligned within 5 Å, highlighted and most common residues involved in binding site were selected. Red color shows binding residues in Gh-CML11 and blue color indicates interacting residues in CLCuMB-βC1. Black arrow represents a closer view of single model among ten models. **B.** Machine learning method PRISM itself evaluated interacting residues within 6 Å for interaction, and interacting amino acids from PRISM are highlighted in red and blue color. **C.** Another machine learning method PAIRPred retrieves data from both sequence and structure. Output result produces graphical illustration of interacting residues between Gh-CML11 and CLCuMB-βC1. Color from orange to red indicates interacting region in both proteins based on B-factor.

We used an ensemble of structure- and sequence-based bioinformatics methods for interface and binding site prediction to identify interacting regions in CLCuMB-βC1 and Gh-CML11 proteins. The individual and ensemble residue-level binding site prediction scores from the bioinformatics analysis are given in S4 Table. Using structural information, protein docking and machine learning methods has identified possible interface site within Gh-CML11 and CLCuMB-βC1 complex (Fig 3A and 3B).

**Fig 4:**
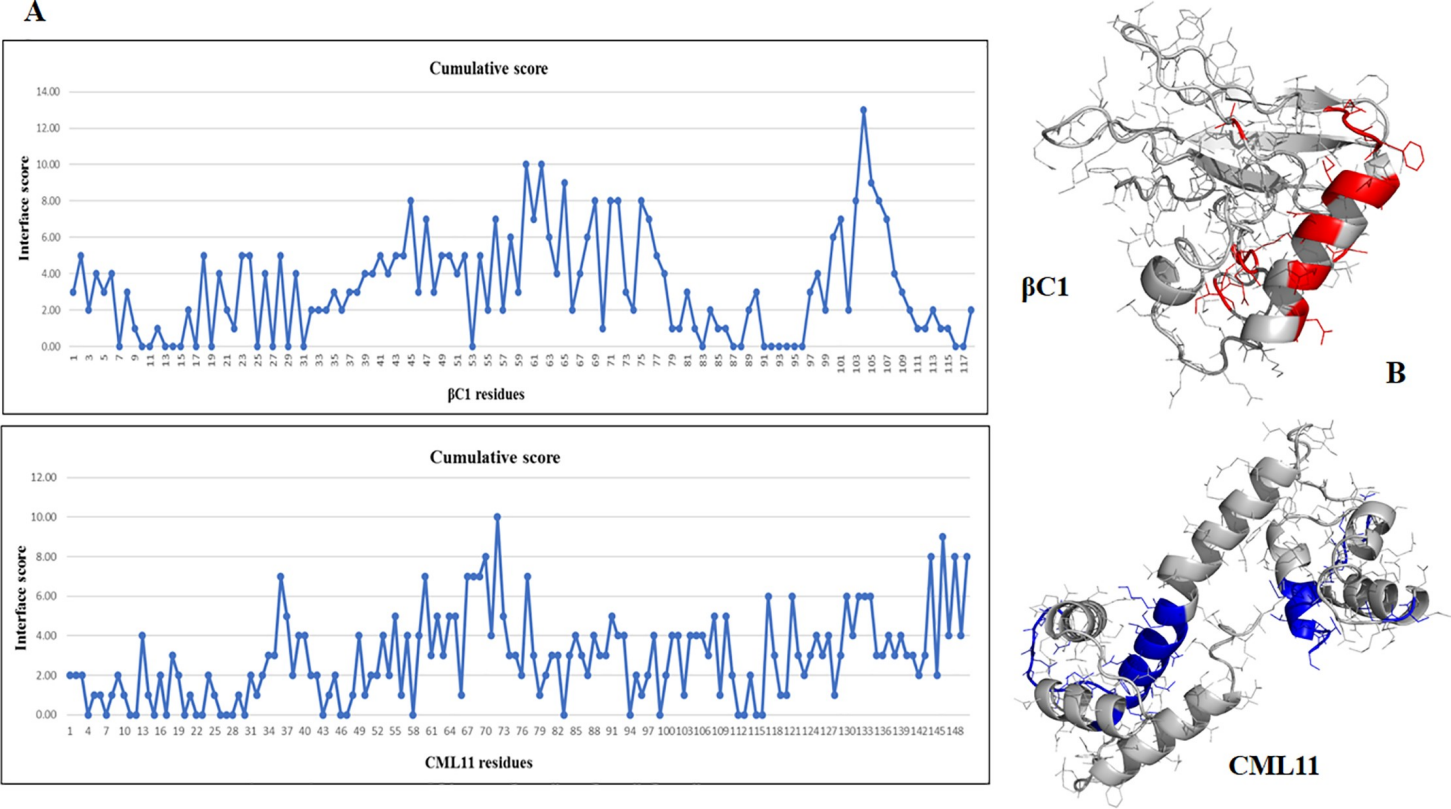
Cumulative score for binding site between CLCuMB-βC1 and Gh-CML11 protein based on sequence and structure prediction methods. **A.** Graphical illustration shows binding site results at residue level. CLCuMB-βC1 residues from 60–75 position in α-helix and residues at 103–106 position has high binding affinity for interaction. Consensus of all computational methods shows Gh-CML11 possess higher binding affinity in the central part (residues at position 60–77) and C-lobe (residues at position 144–150) respectively. **B.** Predicted residues are shown in both virus and host protein structures.

From the cumulative score from S4 Table, it was found that CLCuMB-βC1 possesses two main binding regions: residues 60–75 and 103–106 that form the main α-helix and a loop-turn, respectively, as shown in (Fig 4A). For host protein, linker region (60–77 amino acid) and C-lobe residues (144–150) in Gh-CML11 possess high score for binding (Fig 4A). A known CaMB motif (1-5-8-14) in CLCuMB-βC1 was also a part of this predicted domain at location 66–79, while 103–106 is part of myristylation-like motif 103‘GMDVNE’108. It has already been validated that α-helix and myristylation-like motif in CLCuMB-βC1 are mainly involved in interaction with several host proteins [63]. All predicted regions for virus and host were also localized in their respective 3D structures (Fig 4B). However, deletion of these motifs in both proteins reduces ΔΔG value suggesting the involvement of these motifs in interaction with each other. Thus, computational biology including binding affinity and interaction prediction methods for CLCuMB-βC1 and Gh-CML11 complex provided another approach for exploring the interactions between virus and host proteins.

### Yeast two hybrid (Y2H) and Pull down assay for interaction determination

**Fig 5:**
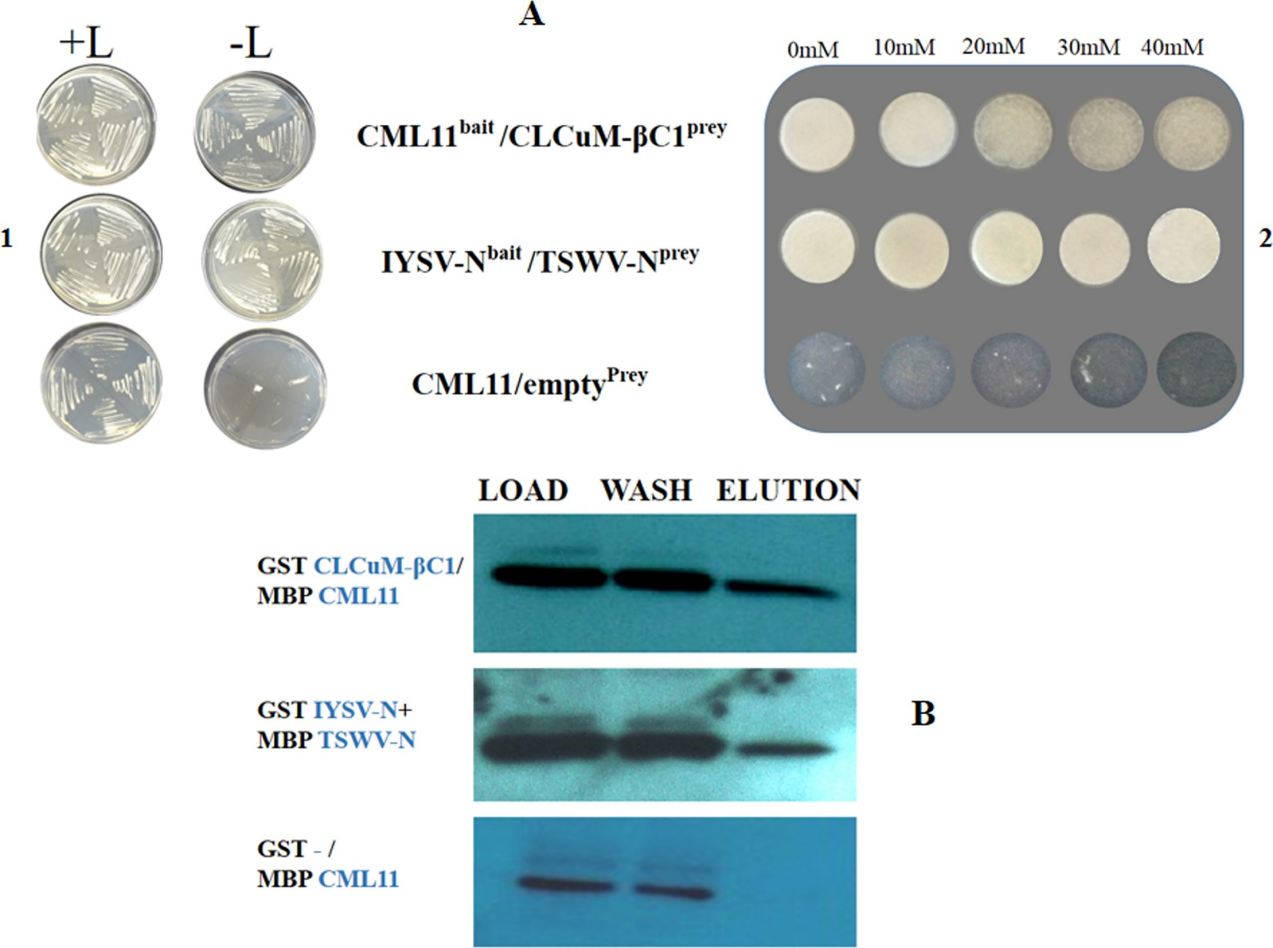
Interaction between Gh-CML11 and CLCuMB-βC1 using yeast two hybrid system and pull down assay. **A.** Positive colonies on yeast agar media [SD-Ura-His-Trp (+L) and SD-Ura-His-Trp-Leu (-L)] indicated interaction between Gh-CML11 and CLCuMB-βC1. Interaction was further enhanced with serial dilution of 3-AT that confirmed the *in-silico* results for Gh-CML11 and CLCuMB-βC1. Interaction of IYSV-TSWV coded N proteins were assessed as a positive control in y2h system. Gh-CML11 fused with empty prey vector was used as a negative control during autoactivation step. **B.** Gh-CML11 protein cloned in pMAL-c2X/MBP was purified with CLCuMB-βC1 protein present in pDEST15/GST-tagged. Purification steps includes load samples that represents crude extracts, wash sample shows removal of unbound proteins and elution samples represents band for positive interaction for Gh-CML11 and CLCuMB-βC1. IYSV-TSWV N proteins were used as a positive control and Gh-CML11 transformation with empty prey was used as a negative control.

To verify the observed *in silico* interactions between virus and host proteins, Y2H was performed using entry clones amplified from Gateway^®^ cloning vector pENTR-D-TOPO. Isolated Gh-CML11 gene was submitted to GenBank after sequence confirmation (MK097275). Confirmed host and virus genes were further expressed in the binary destination vector pEZY202 and pEZY45 as bait and prey, respectively. Gh-CML11 protein was fused to the DBD of bait vector and CLCuMB-βC1 was fused to the TAD of prey vector. After successful transformation of the bait construct in EGY48/pSH18-34 strain, a number of colonies appeared for Gh-CML11 protein on selection media (SD Base/Gal/Raf) supplemented with triple dropout nutrient (-His/-Trp/-Ura) as shown +L in Fig 5A1. Further, yeast cell lines harboring bait construct were co-transformed with prey possessing CLCuMB-βC1 protein. Here, colonies appeared only for CLCuMB-βC1-Gh-CML11 complex. A number of colonies were screened on selection media SD Base/Gal/Raf supplemented with -His/-Leu/-Trp/-Ura as shown in -L in Fig 5A1. Only positive interacting colonies had appeared due to the presence of LEU reporter gene in yeast EGY48 strain.

To determine the strength of interaction in this CLCuMB-βC1-Gh-CML11 complex, a higher concentration of 3-Amino-1,2,4-triazole (3-AT) was added which enhance growth on selection media to confrim the observed interaction [64]. With the serial dilution of 3-AT, several colonies were found on 0-10mM. While on 20-40mM concentration of 3-AT, a fewer number of colonies eliminating false positive results showing strong interaction between CLCuMB-βC1 and Gh-CML11 (Fig 5A2). *Iris yellow spotted virus* (IYSV) and *Tomato spotted wilt virus* (TSWV) encoded nucleoproteins (N) proteins were used as a positive control [60]. IYSV-N interacted strongly with TSWV-N protein in different 3-AT serial dilutions, which confirmed the transformation protocol (Fig 5A2). For negative control, Gh-CML11 was co-transformed with empty prey. Very few or no colonies were observed on selection media as well as in serial dilution of 3-AT. This *in-vivo* Y2H study provided a strong experimental evidence that CLCuMB-βC1 binds with Gh-CML11 during virus infection to modulate calcium source as an energy nutrient for helper virus during CLCuD in cotton.

Results from Y2H were further verified by using Maltose binding protein (MBP) pull down assay. Gateway^®^ entry clones were fused into destination vectors. Gh-CML11 was recombined into pMAL-c2X and CLCuMB-βC1 was fused into pDEST15 vector. In-frame cloning was done in *E*.*coli* BL21 (DE3) strain which was purified using sonication method. Following the Western blotting after protein purification, the nitrocellulose membrane was washed with specific antibodies. Bands were visualized on X-ray film using ECL method. In case of elution sample possessing CLCuMB-βC1-Gh-CML11 complex, a single band was observed with no cross reactivity (Fig 5B). This clear band in elution sample was indicative of the physical interaction of CLCuMB-βC1 and Gh-CML11. MBP-N protein from IYSV and GST-N protein from TSWV were used as a positive control (Fig 5B). A brighter band in the elution sample confirmed the test protocol, while GST alone expressed with MBP-Gh-CML11 developed no band in the elution sample (Fig 5B). These results confirmed the findings from the Y2H assay and proved a strong physical interaction between CLCuMB-βC1 and Gh-CML11 proteins.

### Localization study using bimolecular fluorescence complementation (BiFC) system

**Fig 6:**
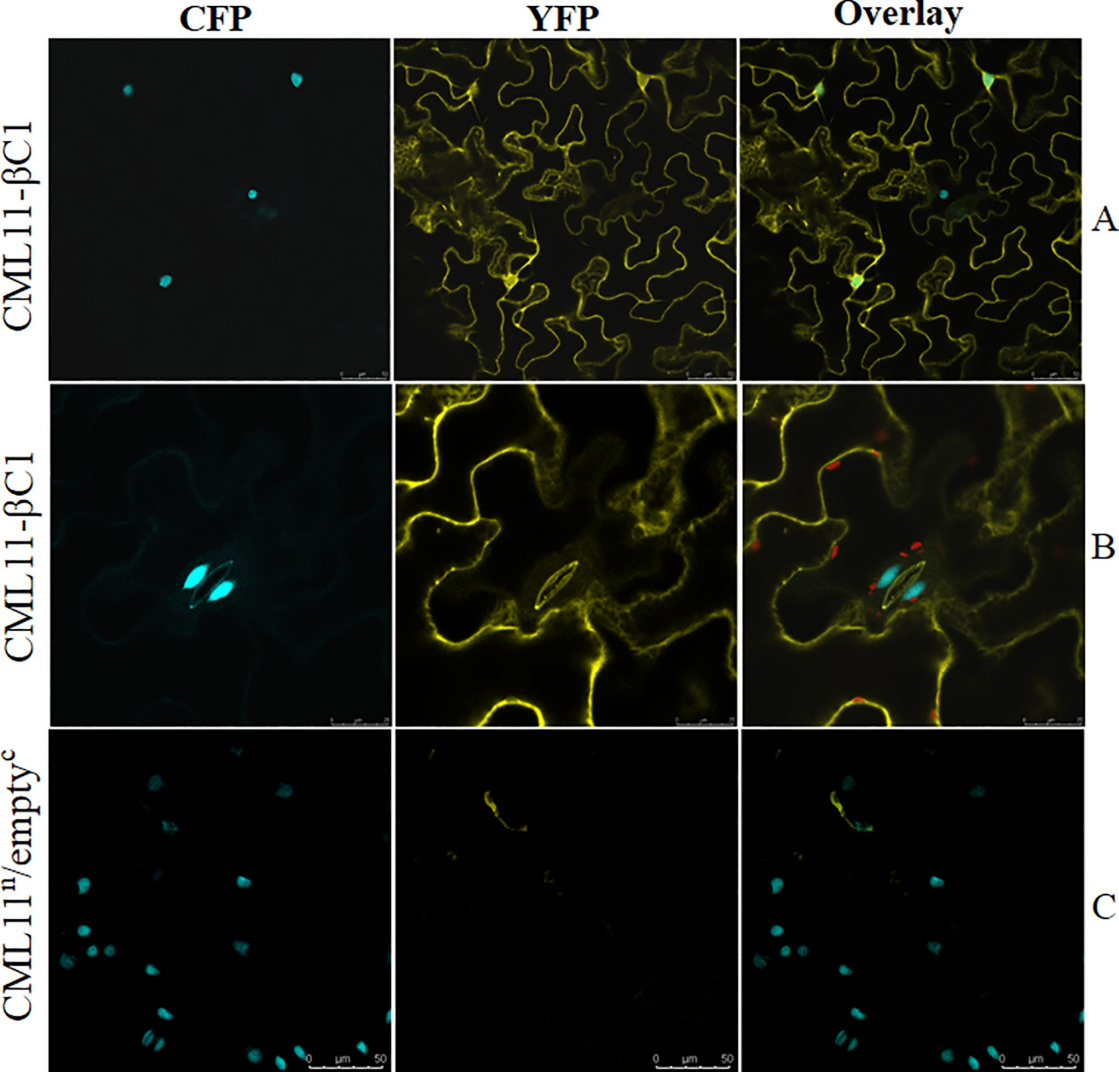
Subcellular localization of Calmodulin like protein Gh-CML11 interaction with CLCuMB-βC1 protein. Gh-CML11 with CLCuMB-βC1 were cloned into GATEWAY compatible BiFC vectors for interaction analysis. For protein localization, *Nicotiana benthamiana* leaves was used, carrying cyan fluorescent protein fused with nuclear specific histone marker 2B (CFP-H2B). **A.** Interaction between Gh-CML11 with CLCuMB-βC1 was localized in plasma membrane. **B.** Guard cells fluorescein with cyan color indicates localization in the cytoplasm. **C.** Negative control showing successful transformation protocol. Images were obtained 48h post-infiltration at 20x+1.5 zoom option. Scale bar = 50 μm.

To determine protein interaction and its localization between CLCuMB-βC1 and Gh-CML11, *in-planta* BiFC assay was performed. Entry clones from pENTR-D-TOPO vector were recombined into binary vector pSITE I series. Gh-CML11 was fused into the N terminal of pSITE-nEYFP-C1 and CLCuMB-βC1 was cloned into the C terminal of pSITE-cEYFP-C1 under 35S promoter. For agroinfiltration, 4 to 5 leaves of *N*. *benthamiana* were infiltrated. After 48 hours, leaf sections were studied under confocal microscopy. We found that CLCuMB-βC1 strongly interacted with Gh-CML11 based on the observation of predominant interaction pattern throughout cytoplasmic veins under yellow fluorescent marker (Fig 6A). To assess the cellular localization, nuclear specific histone 2B fused with CFP marker was used. A clear interaction pattern was found to be distributed throughout nucleus and cytoplasm. At higher magnification (63X), strong signals for guard cells with CLCuMB-βC1 and Gh-CML11 interaction were observed (Fig 6B). The negative control harboring only Gh-CML11 at N terminal with the empty C fragment did not produce any non-specific signals. This test construct expressed very little or zero signal under both YFP and CFP marker (Fig 6C). These results confirmed that CLCuMB-βC1 interact Gh-CML11 interact in nucleus and cytoplasm to stimulate physiological responses for virus movement and transmission.

## Discussion

CML proteins have been shown to play an important role in plant’s immune system against bacterial, fungal and viral pathogens. In plants, three main families of EF-hand Ca^+2^ namely CaM and CML proteins, calcineurin-B like (CBL) proteins and Ca^+2^-dependent protein kinase (CDPK) are known in physiological pathways based on Ca^+2^ sensors. Among all these members, the function(s) of CML proteins are not completely understood in plants, and they do not have much identity in structure with other EF-hand proteins. Recently, data from gene expression profiling and regulatory process in downstream signaling have revealed their function in plants against biotic and abiotic stresses [65]. Here in this study, the role of CML was identified via interaction with CLCuMB-βC1 encoded by a begomovirus associated betasatellite involved in CLCuD in cotton. Based on *in-silico* analysis, it was found that Gh-CML11 possesses a different EF-hand formation that makes it more appropriate for binding with CLCuMB-βC1 in a calcium-dependent manner.

The OW begomoviruses are usually associated with a DNA satellite referred to as betasatellite that encodes a single gene in complementary sense orientation *i*.e βC1 [12]. βC1 is a multifunctional protein which interferes with metabolic and signaling pathway acts as a suppressor of transcriptional gene silencing and post-transcriptional gene silencing [66, 67] and modulates miRNA level during plant development [68]. Viral proteins such as CLCuMV-βC1 can establish mild to severe infection in the host plants using host autophagy machinery. It has been studied that autophagy related genes such as ATG5 and ATG8 in *N*. *benthamiana* helps geminiviruses (CLCuMV, TYLCV and TYLCCNV) to enhance the infectivity in plants. Whereas, silencing of glyceralaldehyde-3-phosphate dehydrogenase (GAPC) gene activates the autophagy process that delayed virus symptoms and producing resistance against CLCuMV [69].

This mode of action mediated by βC1 and other viral protein participates in the accumulation of a high DNA level of helper begomovirus to cause infectivity [70, 71]. In a previous study, it was determined that TYLCCNB-βC1 upregulates NbrgsCaM to mediate symptoms modulation that establishes the successful TYLCCNV infection in Solanaceae hosts [21]. However, low sequence identity of virus coded TYLCCNB with CLCuMB and host coded protein rgsCaM with Gh-CML11, it was essential to determine the role of CML proteins in *G*. *hirsutum* during pathogen attack and to explore the mechanism of CLCuD infection in its natural host, cotton (*G*. *hirsutum*). A higher binding affinity and several canonical CaMB motifs overlapping at different location in CLCuMB-βC1 suggested a strong bonding with Gh-CML11 in nucleus and cytoplasm. CaM binding proteins possess IQ/IQ-like motifs in canonical and non-canonical form and interacts with CAM either in Ca^+2^-dependent or Ca^+2^-independent manner [44]. However, CLCuMB-βC1 possesses canonical forms of CaMB motifs which is an indication that CLCuMB-βC1 could bind Gh-CML11 in a Ca^+2^ dependent manner. Further studies are warranted to validate these results.

The sequence- and structure-based interaction prediction methods showed that the myristylation-like motif of CLCuMB-βC1 ‘GMDVNE’ has a strong binding affinity with Gh-CML11. This hydrophobic region was found to interact with tomato UBC3 protein, showing its importance in the ubiquitination pathway [63]. The role of CLCuMB-βC1 has already been studied in plant ubiquitination pathway. It binds with either SKP1 or CUL1 that impairs the SCF complex during ubiquitination. This impaired binding results into a retarded leaf growth and abnormal development with severe disease symptom [72]. While the host protein, Gh-CML11, possesses the myristylation signal present at 1-MGD-3 (M-G-x), showing the basic difference between calmodulin like protein and calmodulin protein, as true calmodulin proteins lack this myristylation signal. Moreover, calmodulin and calmodulin like protein constitute N- and C-lobes that are connected via Ca^+2^ based EF hands. It has been shown that binding of targeted partners with CML protein at C-lobe is more selective resulting in specific conformational changes in its structure leading to a strong binding [73]. In this study, structure based analysis identified residues that are present at C-terminal lobe showing higher binding affinity for βC1 protein. This computational study suggested that structural biology could predict binding sites between two proteins that offers an efficient and fast-track approach to gain initial insights into all possible interactions of proteins associated with cotton leaf curl disease complex in cotton.

The *in-silico* interaction prediction was verified and confirmed by using molecular techniques that validated the interaction between CLCuMB-βC1 and Gh-CML11. Previous work has shown the localization of TGMV-encoded C2 protein and rgsCaM in cytoplasm and nucleus using YFP and H4-RFP respectively. Notably, TGMV-C2 overexpresses rgsCaM in the nucleus to enhance virus DNA transcription that promote the infection [22]. Therefore, in response to CLCuD, CLCuMB-βC1 binds with Gh-CML11 in cytoplasm and nucleus and this localization was confirmed using YFP and H2B-CFP markers. This interaction indicates that CLCuMB-βC1 is also capable to overexpress Gh-CML11 to induce pathogenicity during CLCuD. Altogether, *in-silico* and molecular approaches have shown the interaction between CLCuMB-βC1 and Gh-CML11 in cotton. Studying biological function of calmodulin protein like (rgsCaM), it acts as endogenous suppressor of RNA silencing during geminivirus infection. It has been determined betasatellite protein βC1 associated with TYLCCNV over expresses the rgs-CaM protein which is required to suppress PTGS pathway and symptom enhancement [21]. This interaction reduces the level of secondary siRNA by repressing RNA dependent RNA polymerase 6 (RDR6) that weakens the plant antiviral response against geminivirus. Another CaM protein in Nicotiana benthamiana does not bind with RDR6 but degrades suppressor of gene silencing 3 (SGS3) in cytoplasm that promotes TYLCCNV. In response to this viral infection, plant also blocks this SGS3 degradation via autophagy inhibitor phosphatidylinositol 3-kinase (PI3K) based on autophagy pathway [74]. Thus, it can be hypothesized that Ca^+2^ ion flux across the cell membrane is being affected after the onset of CLCuMB-βC1 associated with CLCuMV that modulates interaction with CML proteins. This interaction may alter the response of antiviral genes such as RDR6 in *G*. *hirsutum* to enhance the infection. Additional work is required to prove this hypothesis.

## Conclusions and future prospects

Here we have shown that bioinformatics study could reveal interaction information between a geminivirus and its host proteins, and also identify discrimination between CaM and CML protein such as Gh-CML11 proteins based on sequence and structure information. The interaction of CLCuMB-βC1 with Gh-CML11 appears to utilize Ca^+2^ ion channels and pumps to overcome host antiviral defense mechanism, leading to the successful infection and multiplication of CLCuD. This information can be used in future to devise novel strategies to develop resistance against CLCuD. Deletion and/or site-specific mutagenesis in different EF hands in Gh-CML11 would facilitate the identification of residue-based interaction prediction that are directly involved in binding with CLCuMB-βC1. This would establish a more reliable bioinformatics approach for interaction prediction in case of CaM and CML proteins with other plant viruses.

## Supporting information

S1 Table List of primers used in this study. (XLSX)

S2 Table Sequence alignment data of Gh-CML11 with NbrgsCaM and AtCML38. (TXT)

S3 Table Values for binding energy between Gh-CML11 and CLCuMB-βC1. (XLSX)

S4 Table Consensus score for Gh-CML11 and CLCuMB-βC1 to determine interaction using several computational methods. (XLSX)
